# Immunogenicity of vaccination against influenza, *Streptococcus pneumoniae* and *Haemophilus influenzae* type B in patients with multiple myeloma

**DOI:** 10.1054/bjoc.1999.1088

**Published:** 2000-03-06

**Authors:** J D Robertson, K Nagesh, S N Jowitt, M Dougal, H Anderson, K Mutton, M Zambon, J H Scarffe

**Affiliations:** Christie Hospital NHS Trust, Withington, Wilmslow Road, Manchester, M20 4BX, UK; Public Health Laboratory Service, Withington Hospital, Manchester, UK; Central Public Health Laboratory, Colindale, London, UK

**Keywords:** myeloma, influenza, *Streptococcus pneumoniae*, *Haemophilus influenzae*, vaccination

## Abstract

Vaccination against influenza and *Streptococcus pneumoniae* is recommended for elderly and immunocompromised individuals. However, there is little information concerning the efficacy of vaccination in specific groups of patients. In this study, 52 patients underwent vaccination against influenza, *S. pneumoniae* and *Haemophilus influenzae* type b (Hib) as they attended hospital outpatient clinics. Serum was analysed prior to vaccination and 4–6 weeks afterwards. Antibody titres against *S. pneumoniae* and Hib were compared with reference values corresponding to the geometric mean titres of a healthy UK population. For influenza vaccination, haemagglutination inhibition (HI) titres were measured against three inactivated strains; a titre of ≥ 1/40 was considered protective. No patient had protective titres to all three antigens prior to vaccination and 41 patients (85%) had titres < 1/40 to all 3 strains. Post vaccination only 9/48 patients (19%) achieved protective antibody titres. Resistance to *S. pneumoniae* and response to Pneumovax II was also poor: prevaccination, 45 patients (93%) had suboptimal antibody titres and in 26/43 patients (61%) titres remained low post vaccination. Resistance to Hib and response to vaccination was comparable with the healthy adult UK population. These results question the practice of routine influenza and pneumococcal vaccination in myeloma patients. © 2000 Cancer Research Campaign


*British Journal of Cancer* (2000) **82**(7), 1261–1265
© 2000 Cancer Research Campaign
DOI: 10.1054/ bjoc.1999.1088, available online at http://www.idealibrary.com on IDEAL®

### Immunogenicity of vaccination against influenza, *Streptococcus pneumoniae* and *Haemophilus influenzae* type B in patients with multiple myeloma

**JD Robertson[1], K Nagesh[1], SN Jowitt[1], M Dougal[1], H Anderson[1], K Mutton[2], M Zambon[3] and JH Scarffe[1]**

[1]Christie Hospital NHS Trust, Withington, Wilmslow Road, Manchester M20 4BX, UK; [2]Public Health Laboratory Service, Withington Hospital, Manchester, UK; [3]Central Public Health Laboratory, Colindale, London, UK

**Summary** Vaccination against influenza and *Streptococcus pneumoniae* is recommended for elderly and immunocompromised individuals. However, there is little information concerning the efficacy of vaccination in specific groups of patients. In this study, 52 patients underwent vaccination against influenza, *S. pneumoniae* and *Haemophilus influenzae* type b (Hib) as they attended hospital outpatient clinics. Serum was analysed prior to vaccination and 4–6 weeks afterwards. Antibody titres against *S. pneumoniae* and Hib were compared with reference values corresponding to the geometric mean titres of a healthy UK population. For influenza vaccination, haemagglutination inhibition (HI) titres were measured against three inactivated strains; a titre of ≥ 1/40 was considered protective. No patient had protective titres to all three antigens prior to vaccination and 41 patients (85%) had titres < 1/40 to all 3 strains. Post vaccination only 9/48 patients (19%) achieved protective antibody titres. Resistance to *S. pneumoniae* and response to Pneumovax II was also poor: prevaccination, 45 patients (93%) had suboptimal antibody titres and in 26/43 patients (61%) titres remained low post vaccination. Resistance to Hib and response to vaccination was comparable with the healthy adult UK population. These results question the practice of routine influenza and pneumococcal vaccination in myeloma patients. © 2000 Cancer Research Campaign

**Keywords**: myeloma; influenza; *Streptococcus pneumoniae*; *Haemophilus influenzae*; vaccination

Multiple myeloma accounts for approximately 10% of all haematological malignancies. It is a clonal proliferation of plasma cells. In 98% of patients there is a detectable paraprotein (a monoclonal immunoglobulin heavy chain and/or light chain) in the blood or urine. Normal immunoglobulin levels and the ability to mount a humoural response to infection or vaccination are reduced (Fahey et al, 1963). In addition, chemotherapy-induced suppression of the bone marrow and immune system, depression of circulating CD19+ cells, impaired renal function, immobility and, late in the disease, defective cell mediated immunity all contribute to an increased risk of infection in these patients (Deresinski and Stevens, 1974; Norden, 1980; Luque et al, 1998). In the early stages and plateau phase bacterial infections with common organisms such as *Streptococcus pneumoniae*, *Haemophilus influenzae* and *Escherichia coli* predominate (Savage et al, 1982). During the terminal stage of the disease and following chemotherapy or bone marrow transplantation the range of infections increases and includes viruses and fungi (Ringden, 1997). The use of prophylactic agents such as co-trimoxazole, oral antifungal preparations and acyclovir has become standard supportive therapy and has reduced the incidence of infection associated with modern treatment regimens. Despite this, infection remains the leading cause of death in patients with this disease.

*Received 24 May 1999*
*Revised 5 November 1999*
*Accepted 15 November 1999*

*Correspondence to:* JD Robertson

We have undertaken a pilot study in 52 patients with multiple myeloma in order to: (i) estimate the degree of susceptibility of this population to infection with *S. pneumoniae*, *H. influenzae* type b (Hib) and influenza virus and (ii) measure the serological response to three vaccines administered simultaneously.

### MATERIALS AND METHODS

### Patient characteristics

Ethical approval was obtained before commencing this study. Patients attending the weekly myeloma outpatient clinic at the Christie Hospital during October and November 1997 were offered vaccination against influenza, *S. pneumoniae* and *H. influenzae* type b (Hib). Exclusion criteria were allergy to egg, previous influenza vaccine within 6 months, and previous pneumococcal vaccine within 5 years. Written informed consent was required and patients were able to choose whether to receive all or some of the offered vaccines. The vaccines used in the study were: Fluvirin (Evans Medical) containing inactivated A/Singapore/6/86 (H1N1), A/Wuhan/359/95 (H3N2) and B/Beijing/184/93; Pneumovax II (Pasteur-Merieux), a 23-valent vaccine; and ACT-HIB (Farillon). All were administered as deep subcutaneous injections at separate sites according to manufacturers' instructions. Patients were asked to report adverse reactions to vaccination.

Fifty-two patients (30 male, 22 female) were recruited. The median age was 55.4 years (range 31.8–76.4 years). Thirty-nine patients had IgG myeloma, six IgA, one IgD, one systemic AL amyloidosis, four Bence Jones myeloma and one non-secretory myeloma. Eight patients had white blood cell counts below $3.0 \times 10^9\ l^{-1}$ on the day of vaccination. The median granulocyte count of these eight patients was $1.6 \times 10^9\ l^{-1}$. Thirty-five patients had normal levels of polyclonal IgG (local normal range 8–16 g $l^{-1}$) and 17 patients had levels below 8 g $l^{-1}$ on the day of vaccination. In cases of IgG myeloma, this was calculated by subtracting the paraprotein from the total IgG.

Sixteen patients had received chemotherapy within the week prior to vaccination, seven patients had undergone high-dose therapy (melphalan/total body irradiation) and autologous peripheral blood stem cell transplant within the preceding 6 months. Twenty-one patients were undergoing treatment with interferon $\alpha$ 3 MU three times per week during the course of the study.

Forty-seven patients received all three vaccinations and five patients received two of the three offered.

In order to estimate clinical efficacy of influenza vaccination, patients were asked to report episodes of upper respiratory tract infections and given a swab kit with instructions for taking throat and nose swabs for influenza virology.

### Serological methods

Peripheral blood was taken prior to vaccination to estimate baseline serology and at 4–6 weeks following vaccination to measure any increase in specific antibody titres. Serum was stored frozen at –40°C prior to analysis. Anti-capsular polysaccharide antibodies to *S. pneumoniae* and Hib were measured by antigen-specific enzyme-linked immunosorbent assay (ELISA) and radio antigen binding assay (RABA) respectively (Hazelwood et al, 1993) at the Department of Immunology, City Hospital NHS Trust, Birmingham. Antibodies to the three inactivated influenza strains were assayed by haemagglutination inhibition (HI) (Chakraverty 1971) at the Influenza Laboratory, Central Public Health Department, London.

### Interpretation of results

Antibody titres to capsular polysaccharide elements of *S. pneumoniae* and Hib for individual patients were compared with reference values corresponding to the geometric mean titre (GMT) obtained from a healthy UK adult population: 1:640 and 1.02 μg $ml^{-1}$ respectively (Hazelwood et al, 1993). Protective titres were defined as levels above the GMT for these two organisms. For the three influenza antigens, a HI titre of ≥ 1/40 was considered protective for each antigen separately (Hobson et al, 1972). The pre- and post-immunization GMTs allowed further estimation of the population susceptibility and efficacy of vaccination. In order to calculate the GMT for influenza strains, haemagglutination titres below the limit of detection of the assay (1/10) were assigned a value of 5 (Lyall et al, 1997). For individual patients response to vaccination was also determined, a good response being defined as a fourfold increase in antibody titre as a result of vaccination (Zuckerman et al, 1991). Finally, response to individual vaccines was correlated with a number of clinical variables (age, white blood cell count, polyclonal IgG level and current immunosuppressive therapy) using the Pearson Contingency Coefficient. A contingency coefficient of ≥ 0.4 indicated a probable correlation. The significance of the correlation was investigated using the Pearson $\chi^2$ test.

**Table 1** Response to Pneumovax II (total IgG). A protective titre is defined as ≥ 1/640, the geometric mean titre of the normal adult UK population

| | Pre-vaccination (*n* = 48) | | Post-vaccination (*n* = 43) | |
|---|---|---|---|---|
| Protective antibody titre | 3 | 6% | 17 | 39% |
| Suboptimal antibody titre | 45 | 94% | 26 | 61% |
| Titre below 10th percentile | 31 | 70% | 13 | 30% |
| Fourfold increase in titre | | | 24 | 56% |
| Geometric mean titre | 1/53 | | 1/287 | |

**Table 2** Response to ACT-HIB.

| | Pre-vaccination (*n* = 46) | | Post-vaccination (*n* = 44) | |
|---|---|---|---|---|
| Protective antibody titre | 21 | 46% | 33 | 75% |
| Suboptimal antibody titre | 25 | 54% | 11 | 25% |
| Titre < 0.15 μg $l^{-1}$ | 1 | 2% | 0 | |
| Geometric mean titre (μg $l^{-1}$) | 1.06 | | 2.6 | |
| Four fold increase in titre | | | 18 | 41% |

A protective titre is defined as ≥ 1.02 μg $l^{-1}$, the geometric mean titre of the normal UK adult population. The minimum protective level is 0.15 μg $l^{-1}$

### RESULTS

### Specific antibodies to *S. pneumoniae* and response to vaccination (Table 1)

Prior to vaccination, 3/48 patients (6%) had specific anti-pneumococcal capsule polysaccharide antibodies at protective titres (defined as titres greater than 1:640, the geometric mean titre of the normal UK population). Thirty-one patients (70%) had titres below the 10th centile titre of 1:80. Post-vaccination antibody titres were available for 43 patients. Seventeen (40%) achieved protective specific antibodies 4–6 weeks following vaccination. In 26 (61%), however, suboptimal titres were reached, and in 13 patients (30%) antibody titres remained below the 10th centile. A good serological response to vaccination, defined as achieving a fourfold increase in specific antibodies, was recorded in 24 patients (56%). The pre- and post-vaccination GMT for the population were 1:53 and 1:286, indicating that this population as a whole has poor antibody levels against *S. pneumoniae* even after vaccination.

### Specific antibodies to Hib and response to vaccination (Table 2)

Prior to vaccination the antibody titres of the patient population ($n$ = 46) against this organism was comparable with results obtained from the healthy UK adult population. The geometric mean antibody titre was 1.06 μg $l^{-1}$, with 25 patients (54%) having suboptimal levels, and only one patient (2%) having < 0.15 μg $l^{-1}$, the minimum protective level. Following vaccination, paired sera were available for 44 patients. Thirty-three patients (75%) achieved protective antibody titres (> 1.0 μg $ml^{-1}$) and the geometric mean titre increased to 2.6 μg $ml^{-1}$. Eighteen patients (41%) achieved a good response to vaccination in terms of a fourfold increase in antibody titre.

© 2000 Cancer Research Campaign

**Table 3** Response to influenza vaccination

| | Pre-vaccination (*n* = 49) | | Post-vaccination (*n* = 48) | |
|---|---|---|---|---|
| Fully protected | 0 | | 9 | 19% |
| Protected against 0 strains | 41 | 84% | 28 | 59% |
| Protected against 1 strains | 4 | 8% | 6 | 13% |
| Protected against 2 strains | 4 | 8% | 5 | 10% |

A protective antibody titre is defined as a reciprocal HI titre of ≥ 40 for each antigen. Full protection requires protective antibody titres to all three influenza strains.

**Table 4** Pre- and post-vaccination geometric mean titres against three influenza antigens

| | H1N1 | H3N2 | B |
|---|---|---|---|
| Pre-vaccination | 5.6 | 7.2 | 7.2 |
| Post-vaccination | 16.1 | 20.3 | 20.6 |

A titre of over 40 is protective.

### Susceptibility to influenza and response to vaccination (Table 3)

Prior to vaccination, no patients had protective titres to all three influenza strains. Forty-one patients (85%) were susceptible to all the strains, four patients (8.3%) had protection from one strain and four patients (8.3%) had protection from two strains. In a healthy adult population, approximately 50% of subjects would be expected to have protective antibody titres against the two influenza A strains (H1N1 and H3N2) with slightly lower antibody titres to influenza B (unpublished data, PHLS).

Following vaccination, paired sera were available for 48 patients. Twenty-eight patients (58%) had a poor antibody response (HI < 1/40) to all three antigens. Thirteen (27%) patients achieved protective levels against H1N1, 15 patients (31%) achieved protective levels against H3N2 and 15 patients (31%) achieved protective levels against influenza B. Overall, six patients (13%) developed protection against one strain and five patients (10%) developed protection against two strains. Only nine patients (19%), however, developed the required protective titres against all three strains to be considered fully protected against influenza. The pre- and post-vaccination GMTs for each strain were low with none exceeding the levels required for a protective response (Table 4).

### Association of clinical variables with response to vaccination (Table 5)

The responses to Pneumovax, combined influenza strains and vaccination against Hib were correlated with a number of variables using the Pearson Contingency Coefficient as a measure of agreement. The variables tested were: age, white blood cell count, serum polyclonal IgG level, administration of chemotherapy within a week prior to vaccine, treatment with high-dose chemotherapy and autologous peripheral blood stem cell transplant within 6 months prior to vaccine and current treatment with interferon α. Two variables were identified which had possible associations with response to vaccination: undergoing stem cell transplantation within 6 months pre-vaccination correlated with a poor response to Hib vaccine (contingency coefficient 0.431, $P$ = 0.04) and receiving chemotherapy within 7 days pre-vaccination correlated with a poor response to influenza vaccination (contingency coefficient 0.479, NS).

**Table 5** Association of response to vaccination with clinical variables

| Patient variables | Response to vaccination | | | |
|---|---|---|---|---|
| | Pneumovax II | ACT-HIB | | Combined influenza strains |
| Age < 50 | 0.158 | 0.042 | | 0.251 |
| WBC | 0.119 | 0.137 | | 0.274 |
| Serum IgG | 0.134 | 0.077 | | 0.344 |
| Chemotherapy within 7 days | 0.161 | 0.082 | | 0.479 |
| PBSCT within 6 months | 0.258 | 0.431 | *P* = 0.04 | 0.311 |
| Current interferon therapy | 0.047 | 0.085 | | 0.288 |

Contingency coefficient values quoted. Values ≥ 0.4 suggest agreement. *P*-values quoted for positive associations.

### Clinical efficacy

No serious adverse reactions to vaccination were reported. Ten patients reported upper respiratory tract symptoms and sent nose and throat swabs for virus detection during the study period (October 1997–January 1998). No cases of influenza were confirmed.

### DISCUSSION

The policy of the UK Department of Health is to offer annual influenza vaccination as a routine to people with underlying diseases that put them at special risk from this infection (HMSO, 1996). This is based on good epidemiological evidence that immunization of those most at risk reduces hospital admissions and deaths (Ahmed et al, 1995). Similarly, pneumococcal vaccination is recommended for patients over 2 years of age in whom pneumococcal infection is likely to be 'more common and/or dangerous' (HMSO, 1996). However, there is little information concerning the efficacy of these vaccinations in patients with different types of malignancies, whose degree of immune suppression and exposure to cytotoxic drugs varies widely and may affect their ability to mount an immune response. This is especially difficult in the case of pneumococcal vaccination, where there is controversy as to what constitutes the optimum protective antibody level, and antibody responses to only a few of the 23 capsule polysaccharides are measured. In addition there is little information concerning the duration of humoral response to vaccination especially in immunocompromised individuals.

There are no recommendations for Hib immunization in adults and data concerning the susceptibility of immunocompromised populations to infection with this encapsulated organism is scarce. Co-administration of the vaccines at different sites is safe and immunogenic (Honkanen et al, 1996).

In a healthy adult population up to 50% of people have protective titres to circulating influenza A strains and slightly less to influenza B strains (unpublished data, PHLS). Following vaccination, over 70% achieve protective titres against two or three strains (Lorio et al, 1989). Of 48 myeloma patients, only two (4%) had

*© 2000 Cancer Research Campaign*

protective titres to one influenza A strain and three (6%) had protective titres to influenza B pre-vaccination. The population studied is thus highly susceptible to influenza infection. In terms of achieving protective levels of antibodies within 6 weeks, the results of vaccination were disappointing with only 14 patients (29%) achieving protection against two or more strains. Poor antibody responses to influenza vaccination have also been reported in heart transplant patients (Admon et al, 1997). In lung cancer patients, however, the response to influenza vaccine was 78% (Anderson et al, 1999) and in children with acute lymphoblastic leukaemia, titres of ≥ 1/40 were found in over 68% of patients 6 months after immunization (Brydak et al, 1998). In this group, serological responses were higher in samples taken at 6 months post-vaccination compared with those from samples taken at 3 weeks post-vaccination; we intend to further investigate this phenomenon in the myeloma patients. One small randomized study investigated clinical efficacy of influenza vaccination in myeloma patients, using the end point parameters of incidence of upper respiratory tract infections and number of hospital admissions during the study period (Musto and Carotenuto, 1997). The authors concluded that influenza vaccine was effective in myeloma patients although no serological analysis was carried out. In our population, no confirmed cases of influenza were recorded. However, there was only very mild influenza activity in the UK during the winter of 1997–8 (Dedman et al, 1998), and therefore the trial was not really a measure of efficacy. There are no data on the efficacy of two-dose influenza vaccines in myeloma patients but improved responses have not been shown in other patient groups (Miotti et al, 1989; Iorio et al, 1997). Interestingly, the addition of a third dose improved antibody responses in heart transplant patients (Admon et al, 1997).

The degree of susceptibility of our population to infection with *S. pneumoniae* is well demonstrated by the finding that 72% of patients had prevaccination titres below the 10th percentile of the normal adult cohort. The response to vaccination was again disappointing; although 56% developed a fourfold increase in antibody titre, the geometric mean titre remained low and 30% of patients still had antibody titres below the 10th percentile. It is likely that such poor responses to vaccination will also be poorly sustained. Larger studies are required in myeloma patients and in other immunosuppressed groups to address the issue of revaccination. Pneumovax II is intended to be efficacious for 10 years and, under current recommendations, the patients would not be revaccinated within 6 years. Repeat vaccination seems to be safer than previously thought, however, and would seem desirable (Jackson et al, 1999).

Exposure and specific immunity to Hib was comparable to the healthy adult population and there would seem to be no indication for introducing this form of vaccination in these patients.

Some investigators have reported associations between response to vaccination and patient age (Sullivan et al, 1990) and serum IgG level (Gribabis et al, 1994). In this study, small patient numbers precluded detection of significant associations between response to vaccination and the variables considered but recent chemotherapy and autologous peripheral blood stem cell transplant showed positive correlation for poor responses. It is well documented that antibody responses are poor within 2 years of transplant (Parkkali et al, 1996) and generally recommended that vaccinations be given at least 2 weeks prior to chemotherapy or around 6 months after completion of treatment.

### CONCLUSIONS

This study confirms that patients with multiple myeloma are susceptible to infection with *S. pneumoniae* and influenza and demonstrate impaired ability to mount a good humoral response to vaccination. In particular, the extremely poor response to influenza vaccination calls into question the policy of vaccinating patients with myeloma using a conventional single-shot influenza vaccine. This small study was unable to address many other clinical variables that will be of relevance such as vaccination responses in MGUS (monoclonal gammopathy of uncertain significance) or untreated myeloma, and in early versus late disease. Our findings highlight the need for many more studies to further define humoral and cellular vaccination responses in terms of clinical efficacy, magnitude and duration of response in order to define suitable immunization protocols for patients with multiple myeloma. New adjuvants for vaccines and new vaccine delivery systems may offer some hope of enhancing immunopriority in some groups of immunosuppressed individuals.

### ACKNOWLEDGEMENTS

We would like to thank Dr J North, Department of Immunology, City Hospital, Birmingham for advice concerning interpretation of serological results following Pneumovax and Hib vaccination, and Mr J Linsell for his help with the data collation.

### REFERENCES

Admon D, Engelhard D, Strauss N, Goldman N and Zakay-Rones Z (1997) Antibody response to influenza immunization in patients after heart transplantation. *Vaccine* **15**: 1518–1522

Ahmed AH, Nicholson KG and Nguyen van Tam JS (1995) Reduction in mortality associated with influenza vaccination during 1989–90 epidemic. *Lancet* **346**: 591–595

Anderson H, Petrie K, Berrisford C, Charlett A, Thatcher N and Zambon M (1999) Seroconversion after influenza vaccination in patients with lung cancer. *Br J Cancer* **80**: 219–220

Brydak LB, Rokicka-Milewska R, Machala M, Jackowska T and Sikorskå-Fic B (1998) Immunogenicity of subunit trivalent influenza vaccine in children with acute lymphoblastic leukaemia. *Paediatr Infect Dis J* **17**: 126–129

Chakraverty, P (1971) Antigenic relationships between influenza B viruses. *Bull WHO* **45**: 755–766

Dedman DJ, Zambon M, Van Buynder P, Fleming DM, Watson JM and Joseph CA (1998) Influenza surveillance in England and Wales: October 1997 to June 1998. *Commun Dis Public Health* **1**: 244–251

Deresinski SC and Stevens DA (1974) Disseminated herpes simplex in untreated multiple myeloma. Paradox of present concepts of immune defects. *Oncology* **30**: 318–323

Fahey J, Scoggin R, Utz J and Szwed C (1963). Infection, antibody response and gamma globulin components in myeloma and macroglobulinaemia. *Am J Med* **35**: 698–707

Gribabis DA, Panayiotidis P, Boussiotis VA, Hannoun C and Pangalis GA (1994) Influenza virus vaccine in B-cell chronic lymphocytic leukaemia patients. *Acta Haematol* **91**: 115–118

Hazelwood M, Nusrat R, Kumararatne DS, Goodall M, Raykundalia C, Gong Wang D, Joyce HJ, Milford-Wards A, Forte M and Pahor A (1993) The acquisition of anti-pneumococcal capsular polysaccharide, *Haemophilus influenzae* type b and tetanus toxoid antibodies, with age, in the UK. *Clin Exp Immunol* **93**: 1–8

HMSO (1996) *Immunisation against Infectious Disease*. Department of Health: London

Hobson D, Curry RL, Beare AS and Ward-Gardner A (1972) The role of serum haemagglutination-inhibiting antibody in protection against challenge infection with influenza A2 and B viruses. *J Hygiene Lond* **70**: 767–777

Honkanen PO, Keistinen T and Kivela SL (1996) Reactions following administration of influenza vaccine alone or with pneumococcal vaccine to the elderly. *Arch Intern Med* **156**: 205–206

*© 2000 Cancer Research Campaign*

Iorio AM, Alatri A, Francisci D, Preziosi R, Neri M, Donatelli I, Castrucci MR, Biasio LR, Tascini C, Iapoce R, Pierucci P and Baldelli F (1997) Immunogenicity of influenza vaccine (1993–94 winter season) in HIV-seropositive and -seronegative ex-intravenous drug users. *Vaccine* **15**: 97–102

Jackson LA, Benson P, Sneller VP, Butler JC, Thompson RS, Chen RT, Lewis L S, Carlone G, De Stefano F, Holder P, Lezhava T and Williams WW (1999) Safety of revaccination with pneumococcal polysaccharide vaccine. *J Am Med Assoc* **281**: 243–248

Lorio AM, Rivosecchi P, Zei M, Neri M, Merlett L (1989) Immune response to trivalent inactivated influenza vaccine in young and elderly subjects. *Vaccine* **7**: 303–306

Luque R, Brieva JA, Moreno A, Manzanal A, Escribano L, Villarrubia J, Velasco JL, Lopez-Jimenez J, Cervero C, Otero MJ, Martinez J, Bellas C and Roldan (1998) Normal and clonal B lineage cells can be distinguished by their differential expression of B cell antigens and adhesion molecules in peripheral blood from multiple myeloma (MM) patients–diagnostic and clinical implications. *Clin Exp Immunol* **112**: 410–418

Lyall EGH, Charlett A, Watkins P and Zambon M (1997) Response to influenza virus vaccine in vertical HIV infection. *Arch Dis Childhood* **76**: 215–218

Miotti PG, Nelson KE, Dalabetta GA, Farzadegan H, Margolock J and Clements M L (1989). The influence of HIV on antibody responses to a two-dose regimen of influenza vaccine. *J Am Med Assoc* **262**: 779–783

Musto P and Carotenuto M (1997) Vaccination against influenza in multiple myeloma (letter). *Br J Haematol* **97**: 505–506

Norden CW (1980) Infections in patients with multiple myeloma [editorial]. *Arch Intern Med* **140**: 1150–1151

Parkkali T, Kayhty H, Ruutu T, Volin L, Eskola J and Ruutu P (1996) A comparison of early and late vaccination with *Haemophilus influenzae* type b conjugate and pneumococcal polysaccharide vaccines after allogeneic BMT. *Bone Marrow Transplant* **18**: 961–996

Ringden O (1997) Allogeneic bone marrow transplantation for hematological malignancies controversies and recent advances. *Acta Oncol* **36**: 549–564

Savage DG, Lindenbaum J and Garrett TJ (1982) Biphasic pattern of bacterial infection in multiple myeloma. *Ann Intern Med* **96**: 47–50

Sullivan KM, Monto AS and Foster DA (1990) Antibody response to inactivated influenza vaccines of various antigenic concentrations. *J Infect Dis* **161**: 333–333

Zuckerman MA, Wood J, Chakraverty P, Taylor J, Heath AB, Oxford JS (1991) Serological responses in volunteers to inactivated trivalent subunit influenza vaccine: antibody reactivity with epidemic influenza A and B strains and evidence of a rapid immune response. *J Med Virol* **33**: 133–137

© 2000 Cancer Research Campaign

## References

[PDF_00397] Admon D., Engelhard D., Strauss N., Goldman N., Zakay-Rones Z. (1997). Antibody response to influenza immunization in patients after heart transplantation.. Vaccine.

[PDF_00400] Ahmed A. E., Nicholson K. G., Nguyen-Van-Tam J. S. (1995). Reduction in mortality associated with influenza vaccine during 1989-90 epidemic.. Lancet.

[PDF_00403] Anderson H., Petrie K., Berrisford C., Charlett A., Thatcher N., Zambon M. (1999). Seroconversion after influenza vaccination in patients with lung cancer.. Br J Cancer.

[PDF_00406] Brydak L. B., Rokicka-Milewska R., Machała M., Jackowska T., Sikorska-Fic B. (1998). Immunogenicity of subunit trivalent influenza vaccine in children with acute lymphoblastic leukemia.. Pediatr Infect Dis J.

[PDF_00409] Chakraverty P. (1971). Antigenic relationship between influenza B viruses.. Bull World Health Organ.

[PDF_00411] Dedman D. J., Zambon M., Buynder P. V., Fleming D. M., Watson J. M., Joseph C. A. (1998). Influenza surveillance in England and Wales: October 1997 to June 1998.. Commun Dis Public Health.

[PDF_00414] Deresinski S. C., Stevens D. A. (1974). Disseminated herpes simplex in untreated multiple myeloma. Paradox of present concepts of immune defects.. Oncology.

[PDF_00417] FAHEY J. L., SCOGGINS R., UTZ J. P., SZWED C. F. (1963). INFECTION, ANTIBODY RESPONSE AND GAMMA GLOBULIN COMPONENTS IN MULTIPLE MYELOMA AND MACROGLOBULINEMIA.. Am J Med.

[PDF_00420] Gribabis D. A., Panayiotidis P., Boussiotis V. A., Hannoun C., Pangalis G. A. (1994). Influenza virus vaccine in B-cell chronic lymphocytic leukaemia patients.. Acta Haematol.

[PDF_00445] Gross P. A., Quinnan G. V., Weksler M. E., Setia U., Douglas R. G. (1989). Relation of chronic disease and immune response to influenza vaccine in the elderly.. Vaccine.

[PDF_00428] Hobson D., Curry R. L., Beare A. S., Ward-Gardner A. (1972). The role of serum haemagglutination-inhibiting antibody in protection against challenge infection with influenza A2 and B viruses.. J Hyg (Lond).

[PDF_00431] Honkanen P. O., Keistinen T., Kivelä S. L. (1996). Reactions following administration of influenza vaccine alone or with pneumococcal vaccine to the elderly.. Arch Intern Med.

[PDF_00436] Iorio A. M., Alatri A., Francisci D., Preziosi R., Neri M., Donatelli I., Castrucci M. R., Biasio L. R., Tascini C., Iapoce R. (1997). Immunogenicity of influenza vaccine (1993-94 winter season) in HIV-seropositive and -seronegative ex-intravenous drug users.. Vaccine.

[PDF_00442] Jackson L. A., Benson P., Sneller V. P., Butler J. C., Thompson R. S., Chen R. T., Lewis L. S., Carlone G., DeStefano F., Holder P. (1999). Safety of revaccination with pneumococcal polysaccharide vaccine.. JAMA.

[PDF_00448] Luque R., Brieva J. A., Moreno A., Manzanal A., Escribano L., Villarrubia J., Velasco J. L., López-Jiménez J., Cerveró C., Otero M. J. (1998). Normal and clonal B lineage cells can be distinguished by their differential expression of B cell antigens and adhesion molecules in peripheral blood from multiple myeloma (MM) patients--diagnostic and clinical implications.. Clin Exp Immunol.

[PDF_00454] Lyall E. G., Charlett A., Watkins P., Zambon M. (1997). Response to influenza virus vaccination in vertical HIV infection.. Arch Dis Child.

[PDF_00457] Miotti P. G., Nelson K. E., Dallabetta G. A., Farzadegan H., Margolick J., Clements M. L. (1989). The influence of HIV infection on antibody responses to a two-dose regimen of influenza vaccine.. JAMA.

[PDF_00460] Musto P., Carotenuto M. (1997). Vaccination against influenza in multiple myeloma.. Br J Haematol.

[PDF_00462] Norden C. W. (1980). Infections in patients with multiple myeloma.. Arch Intern Med.

[PDF_00464] Parkkali T., Käyhty H., Ruutu T., Volin L., Eskola J., Ruutu P. (1996). A comparison of early and late vaccination with Haemophilus influenzae type b conjugate and pneumococcal polysaccharide vaccines after allogeneic BMT.. Bone Marrow Transplant.

[PDF_00468] Ringdén O. (1997). Allogeneic bone marrow transplantation for hematological malignancies--controversies and recent advances.. Acta Oncol.

[PDF_00470] Savage D. G., Lindenbaum J., Garrett T. J. (1982). Biphasic pattern of bacterial infection in multiple myeloma.. Ann Intern Med.

[PDF_00472] Sullivan K. M., Monto A. S., Foster D. A. (1990). Antibody response to inactivated influenza vaccines of various antigenic concentrations.. J Infect Dis.

[PDF_00423] Vladutiu A. O. (1993). The severe combined immunodeficient (SCID) mouse as a model for the study of autoimmune diseases.. Clin Exp Immunol.

[PDF_00474] Zuckerman M. A., Wood J., Chakraverty P., Taylor J., Heath A. B., Oxford J. S. (1991). Serological responses in volunteers to inactivated trivalent subunit influenza vaccine: antibody reactivity with epidemic influenza A and B strains and evidence of a rapid immune response.. J Med Virol.

